# Rewiring gene circuits to dissect oscillatory signaling dynamics

**DOI:** 10.1101/gad.353319.125

**Published:** 2026-01-01

**Authors:** Marek J. van Oostrom, Katharina F. Sonnen

**Affiliations:** Hubrecht Institute-KNAW (Royal Netherlands Academy of Arts and Sciences), University Medical Center Utrecht, Utrecht 3584 CT, The Netherlands

**Keywords:** NOTCH signaling, optogenetics, organoid, segmentation clock, synthetic biology

## Abstract

Precise intercellular communication is critical for cellular decision-making. The segmentation clock is an oscillatory gene network regulating periodic segmentation of the presomitic mesoderm (PSM) in vertebrate embryos. Oscillations between neighboring cells are thought to be coupled by DELTA–NOTCH signaling. To directly test this experimentally, Isomura and colleagues (doi:10.1101/gad.352538.124) reconstituted this coupling using synthetic biology. They integrated a synthetic DELTA–NOTCH pathway into DELTA-deficient PSM organoids, which restored cell–cell communication. Additionally, optogenetic activation of the synthetic ligand further revealed that the dynamics of ligand presentation are crucial for effective communication. This work directly demonstrates the importance of oscillatory cell–cell signaling in development and provides a blueprint for using synthetic circuits in future studies.

## Abstract (precis)

In this Outlook, van Oostrom and Sonnen discuss a study in this issue of *Genes & Development* that uses an optogenetic approach to study oscillatory intercellular communication through synthetic ligand–receptor pairs during segmentation clock synchronization. They highlight the value of such tools in studying or even reprogramming developmental signaling and gene expression dynamics, which would have implications for regenerative medicine and synthetic embryology.

Multicellular development depends on reliable intercellular communication to coordinate fate decisions and tissue patterning. However, intercellular cues can be degraded by noise and diffusion limits, raising the question of how precise information spreads across a tissue. One strategy is to use oscillatory or pulsatile signals that are synchronized among neighbors: By aligning rhythmic pulses, cells achieve robust tissue-wide coordination (Casani-Galdon and Garcia-Ojalvo 2022). Bacterial communities and social amoebae, for example, synchronize internal oscillators to enable collective behaviors.

A prime example is the vertebrate segmentation clock, a genetic oscillator in the presomitic mesoderm (PSM) that controls the periodic formation of somites, precursors of vertebrae and adjacent tissues. Neighboring PSM cells typically maintain coherent albeit slightly phase-shifted oscillations via DELTA–NOTCH signaling. When DELTA signaling is perturbed (e.g., in *Dll1* mutant embryos), tissue-level coordination is impaired, and somite boundaries form abnormally (de Angelis et al. 1997). Despite this, it has remained unclear whether NOTCH ligand–receptor interactions between neighboring cells directly synchronize cellular oscillations and whether the oscillatory nature of DELTA–NOTCH itself confers a synchronization advantage over a static signal.

In this issue of *Genes & Development*, Isomura et al. (2026) address these questions with a synthetic biology approach that reconstructs a minimal cell–cell signaling system and test how dynamic stimuli entrain the segmentation clock between neighboring cells. Such synthetic circuits clarify minimal requirements and enable precise tuning and perturbation, as shown previously for morphogen gradient formation in the *Drosophila* wing primordium (Stapornwongkul et al. 2020). Building on the synNOTCH system of synthetic NOTCH signaling (Morsut et al. 2016), the investigators engineered a synthetic ligand–receptor pair with new specificity (Fig. 1). As the receptor (exNOTCH), the investigators used NOTCH1 with its extracellular domain replaced by an anti-mCherry nanobody. As the ligand (synDELTA), they used a membrane-tethered mCherry fusion that binds exNOTCH. Contact between a synDELTA “sender” cell and an exNOTCH “receiver” cell activates the receptor, releasing the NOTCH intracellular domain (NICD) to drive downstream expression. As a readout, they applied a NOTCH-responsive luciferase reporter (Yoshioka-Kobayashi et al. 2020). In addition, Isomura et al. (2026) implemented a dynamic approach to periodically induce expression, which can typically be achieved by optogenetics or microfluidics (Sonnen et al. 2018). Here, blue-light pulses periodically induced synDELTA in sender cells, producing oscillatory ligand bursts; adjacent receivers expressed exNOTCH and the reporter to record response timing.

**Figure 1.**
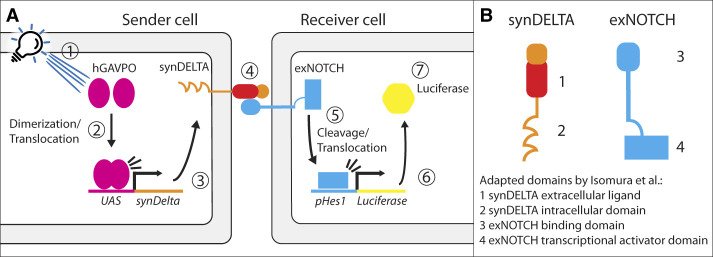
Schematic of the synthetic sender–receiver circuit and engineered synDELTA/exNOTCH proteins. (*A*) Blue-light illumination of sender cells (1) induces hGAVPO dimerization and nuclear translocation (2), activating expression of the synDELTA ligand (3). synDELTA on sender cells engages exNOTCH on receiver cells (4), triggering proteolytic cleavage and nuclear translocation of the exNOTCH intracellular domain (exNICD) (5). exNICD drives a luciferase reporter (6), rendering receiver cells luminescent (7). (*B*) Domain organization and modular engineering of synDELTA and exNOTCH. The extracellular domains of synDELTA (1) and exNOTCH (3) were varied to tune ligand–receptor binding affinity and assess downstream effects. The intracellular domain of synDELTA (2) was engineered to examine its impact on receptor trafficking. The exNOTCH intracellular domain (4) was exchanged for alternative transcriptional activators to couple the pathway to canonical NOTCH signaling or orthogonal reporters (e.g., a cleavable Tet *trans*-activator).

The investigators used the modularity of the system to customize the receptors and thereby the signaling dynamics. Swapping different intracellular domains into the ligand or receptor altered subcellular trafficking and dynamics without changing receptor recognition. Several synDELTA variants with distinct cytoplasmic tails were tested. A synDELTA with the native DLL1 intracellular region relayed signals with a brief delay, whereas the original synNOTCH ligand (lacking DELTA motifs) produced a slower rise in receiver response. An unrelated intracellular tail (from EGF) yielded an intermediate ∼1 h delay. Thus, the ligand's intracellular domain strongly influences cell–cell transmission speed, likely via effects on trafficking or turnover.

Importantly, only the fast-transmitting ligand variants could keep up with high-frequency oscillatory stimulation. When light pulses with a 2 h period were used, receivers showed clear oscillatory reporter activity only when expressing a subset of custom ligands. Even at a 6 h period, slow ligands produced weaker amplitudes than faster ones. These results indicate that ligand presentation kinetics set an upper frequency limit for synchronizable signals and modulate receiver amplitude.

The investigators next integrated the synthetic system into a more complex setting: an optimized, self-organizing PSM organoid platform. Mass induced PSM (miPSM) cultures, derived from embryonic stem cells, recapitulate segmentation clock oscillations and can be produced in large quantities (Matsumiya et al. 2018). Isomura et al. (2026) generated organoids from *Dll1* knockout ESCs (lacking the native DELTA ligand), which show impaired clock oscillations and disrupted somite patterning. They introduced exNOTCH and drove synDELTA under an oscillatory clock promoter, effectively rewiring the circuit to operate through the synthetic pair.

Discriminating between tissue-wide damping and loss of synchrony requires quantification of signaling in single neighboring cells. Importantly, using single-cell quantification, the investigators showed that the synthetic oscillatory pathway could partially restore collective oscillations in *Dll1*-deficient organoids. However, the partial rescue of collective signaling behavior did not result in proper patterning of somite structures downstream from the signaling. These findings demonstrate that synthetic oscillatory cell–cell signaling (synDELTA–exNOTCH) is sufficient to resynchronize the segmentation clock in this system. Additional regulatory features are needed to recover full patterning, which has to be uncovered in the future.

Beyond somitogenesis, this work provides a general model for probing and programming developmental timing. It reveals that the dynamic character of a pathway (specifically, intracellular ligand dynamics) can dictate oscillation timing and the synchronization of cellular oscillators. It also offers the first experimental confirmation that oscillatory coupling provides a synchronization benefit that static signaling does not.

Researchers in signaling dynamics need tools to modulate pathway timing in a controlled manner (Purvis and Lahav 2013). The ability to mix and match ligand and receptor components, especially combined with optogenetics, furnishes a toolkit to tune gene oscillation frequency and amplitude with clear readouts. From a broader perspective, the synthetic “rewiring” of DELTA–NOTCH used here could extend to other biological oscillators. Hes gene dynamics downstream from NOTCH signaling have been implicated in fate decisions across numerous stem cell systems (e.g., neurons, pancreas, muscle, and small intestine) (Chandel et al. 2024; Weterings et al. 2024). In the long term, programming custom oscillatory circuits in stem cell-derived tissues may open avenues in regenerative medicine and synthetic embryology. The study by Isomura et al. (2026) demonstrates the potential of synthetic biology not only to decipher developmental mechanisms but also to actively control them—synchronizing cells at will to build organized, rhythmic patterns of gene activity.
